# Cybergonomics: Proposing and justification of a new name for the ergonomics of Industry 4.0 technologies

**DOI:** 10.3389/fpubh.2022.1012985

**Published:** 2022-11-03

**Authors:** Mostafa Pouyakian

**Affiliations:** Department of Occupational Health and Safety Engineering, School of Public Health and Safety, Shahid Beheshti University of Medical Sciences, Tehran, Iran

**Keywords:** cybergonomics, ergonomics, Industry 4.0, cyber-technologies, human factors

## Abstract

Various subdisciplines of ergonomics science have emerged during the past decades as our insight has been broadened of human and performance. The three main branches of ergonomics have evolved over time focusing on the physical, cognitive, and organizational aspects. But the question is, can these disciplines focus and research enough on the ergonomic aspects of cyberspace and Industry 4.0 technologies? Cyber-technologies of the Fourth Industrial revolution are dramatically imposing themselves on our life and work. This has been led to emerging cyber-aspects for human work and life. Hence, many sciences, mainly applied ones, have upgraded to their cyber versions to deal with the emergent issues, usually with a new name, including the cyber prefix. Cyber-medicine, cyber-health, cyber-commerce, and cyberpsychology are some examples. Also, ergonomics requires a similar look. Ergonomic benefits and threats of Industry 4.0 technologies must be considered in an integrated manner. This paper addresses this issue. First, the emergence and development of ergonomics and its subdisciplines chronologically is reviewed. Then, Cybergonomics as a new name and concept is proposed and defined as the ergonomics of Industry 4.0 era. Justification for this portmanteau is described, and an outline of the new realm is explained. Finally, a research road map is proposed for this new subdiscipline of ergonomics.

## Introduction

### A short history of ergonomics from the perspective of industrial revolutions

Ergonomics/Human factors is a science created and developed in response to the requirements of the industrial revolutions. In other words, the history of ergonomics should be tracked along with the history of technology. First Industrial Revolution (1760–1870) technologies were based on the control of steam power and steam engines. These technologies expanded the new industries like textile factories and railroad transportation in the first half of the nineteenth century. This new situation gradually created new types of work and work environments. In 1857, the last years of the First Industrial Revolution, Wojciech Jastrzebowski (1799–1882) defined a new scientific system to point out the need for a new scientific look at the relationship between humans and work. He wrote a paper and named this new science called ergonomji (in the Polish language), consisting of the two Greek words: Ergon (work) and Nomos (law) (1). The principles of this new knowledge were evolved during the Second Industrial Revolution (1870–1969), where the usage of electricity resulted in establishing the industrial mass production lines. Scientists like Frederick W. Taylor, Frank, and Lilian Gilbreth, at the beginning of the twentieth century, began to lay the foundations of the fundamental principles of human performance relevant to the design and evaluation of industrial systems (2). Their pioneering works on industrial efficiency have a significant contribution to improving mass production.

However, until 1949, ergonomics was not recognized as an independent scientific discipline. In that year, British psychologist “Hywel Murell” (1908–1984) redefined the word ergonomics as a newborn scientific discipline and proposed the official use of the word in the English lexicon (1). After that, this word was widely used by researchers worldwide, and national associations of ergonomics were gradually formed in various countries. The International Ergonomics Association (IEA) was founded as a federation of ergonomics societies in 1959, shortly before the Third Industrial Revolution inauguration (1969–2000). These efforts led to the rapid development of ergonomics and its subdisciplines at the beginning of the Third Industrial Revolution. The invention of computers and the Internet, development of robotics and automation characterized the Third Industrial Revolution. Ergonomics as an interdisciplinary science was developed by studies to cover the increasing need to optimize human performance in this period. The military applications were the powerful driving force to develop this new science. American psychologist, Alphonse Chapanis (1917–2002) was one of the pioneers in this field who strengthened the foundations of new ergonomics science with his innovative studies (3–5). The IEA's historian, Prof. Brian Shackel, has noted the international developmental focus of ergonomics in the second half of the twentieth century as follows: In the 1950s, military ergonomics; in the 1960s, industrial ergonomics; in the 1970s, ergonomics of consumers and goods services; in the 1980s, computer ergonomics; in 1990s, macro and cognitive ergonomics (6).

The Fourth Industrial Revolution (Industry 4.0) was started in the 2000s and characterized by new emerging technologies powered by a new generation of information technology and digitalization. This article will return to these technologies later. Ergonomics is opening up new avenues and areas of research based on emerging technologies of Industry 4.0. Thus, the journey of ergonomics has been accompanied by the evolution of definitions and subdisciplines of this science from the past to the present.

### Evolution of definition and subdisciplines of ergonomics

The definition of ergonomics has evolved along with the historical periods that ergonomics has gone through. The meaning of the word ergonomics, as intended by Jastrzebowski, is the natural laws of work. However, the definition of this science has undergone many changes over time due to its multidisciplinary identity. Definitions are presented from different points of view, from engineering to psychology and from management to physiology.

These definitions are from short dictionary type definitions to long-length definitions of the field. Dempsey et al. (2) analyzed nearly 100 definitions of word ergonomics from various sources and extracted the key and frequent words used to define ergonomics. They formulate their definition in a simple structure of Who (human, people, user, person), What (system, machine, equipment, product, technology), How (engineering, designing, applying, studying, optimizing), When/Where (environment, work, life), and Goal (safety, confront, efficiency) to show the core concepts of the ergonomics science (2). They suggested five moderate-length statements based on frequent words to describe the field. However, their work did not go beyond the definition of ergonomics itself and did not cover the subfields of this science.

**Figure 1:**
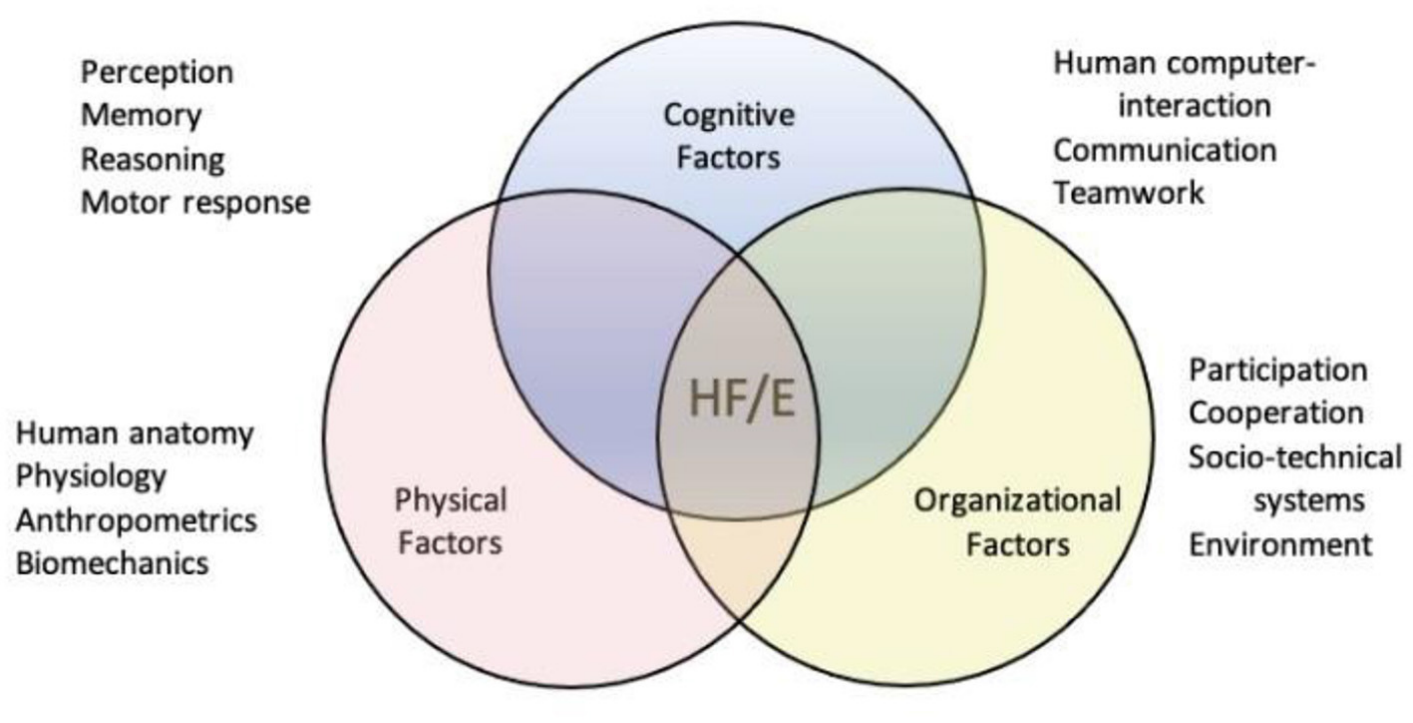
Interdisciplinary identity of ergonomics and the three main classic areas of research (7).

IEA defines Ergonomics as a scientific discipline concerned with understanding interactions among humans and other elements of a system and the profession that applies theory, principles, data, and methods to design to optimize human well-being and overall system performance (7). However, this definition is not directly usable for applied reasons. Hence, IEA has categorized the various factors regarding the aim of ergonomics to clarify the particular subdisciplines of ergonomics (Figure 1). Based on this, the three available subfields of ergonomics are identified as follows: (i) Physical ergonomics, (ii) Cognitive ergonomics, (iii) Systems ergonomics (Macroergonomics). This classification refers to the assessment of human performance in physical, mental, and organizational aspects (8). These subfields of ergonomics were introduced and recognized before 2000. Hollnagel explained the differences and boundaries between cognitive and classical ergonomics (physical ergonomics) in a paper in 1997 (9).

In the mid-1980s, with the development of complex socio-technical systems (STS), the term “macroergonomics” was coined to address the organizational aspects of ergonomics. That is why it is sometimes called “system ergonomics.” This gradually led to the emergence of a new branch of research in human factors in the following decades. This subdiscipline of ergonomics refers to the field of science that concentrates on designing overall work systems by providing the knowledge and methods necessary for improving work systems and, thus, developing the effectiveness and performance of companies (6). Prof. Hal Hendrick was one of the founders of this subdiscipline.

Prof. Raja Parasuraman, in 2003 published a brilliant paper in Theoretical Issues in Ergonomics Science Journal and introduced a new area of applied researches in ergonomics. He coined a new term, neuroergonomics, to address a new branch of ergonomics science (10). This ergonomics subfield merges the disciplines of “traditional” neuroscience and “conventional” ergonomics to maximize the benefits of each. While traditional neuroscience studies the function and structure of the nervous system, neuroergonomics provides added value to understanding brain function and behavior as it occurs in the real world (11).

Along with these well-known subfields of ergonomics, some other branches have been introduced over the years, like participatory ergonomics (12) and cultural ergonomics (13). Also, Zink and Fischer (14) considered the sustainability paradigm and the opportunities that this paradigm can be created for new areas of research in ergonomics. They proposed general principles for designing new and enhancing human factors and ergonomics approaches regarding their orientation toward the sustainability paradigm.

The names of the subfields of ergonomics represent the focus of each subfield on human performance. Physical, mental, and organizational or system aspects of human performance lie in the names of the subfields mentioned above. It helps researchers to focus their works on their desired aspects. Also, evolving these names shows a chronological fact related to the gradual development of technologies that forces the movement from physical ergonomics to systems ergonomics and, recently, cognitive and neuroergonomics. Cyber-identity is the newest aspect of humans. Thus, it is time to go forward toward a new type of ergonomics.

### Industry 4.0 technologies

The German government used the concept of Industry 4.0 in 2011 to refer to the next generation of the computerization and emergence of new technologies. Klaus Schwab, founder and executive chairman of the World Economic Forum, published a book (Klaus Schwab, The Fourth Industrial Revolution; Crown, 2017) explaining the specifics of this new industrial revolution. Emerging technologies such as 3D printers, self-driving cars, smart cities and organizations, smart medicine and healthcare, ubiquitous computing, next-generation robotics, blockchain, artificial intelligence, wearable internet, and implantable equipment are gradually evolving and commercialized (15). According to Schwab, drivers of the Industry 4.0 technologies are (i) physical, (ii) digital, and (iii) biologic. Autonomous vehicles, 3D printing, advanced robotics, and new/smart materials are the most striking emergence of physical drivers. Internet of Things (IoT) and blockchain technology are two impressive manifestations of digital drivers. Finally, bioprinting, gene sequencing and editing, synthetic biology, and precision medicine represent the biological drivers of Industry 4.0 technologies. Other classifications have also been proposed for Industry 4.0 technologies. Boston Consulting Group has been proposed a classification, including nine classes: additive manufacturing, augmented reality, vertical and horizontal data integration, simulation, cyber security, industrial internet of things, big data analytics, cloud computing, and human-robot collaboration (16). Briefly, the world of Industry 4.0 can be described by some words like hyper-connected technologies and people, complexity, smart devices, and the smart world, ambient and cloud computing, intelligent assistants, sensors, cyber-physical systems, implanted technologies, smart textiles, and wearables. Regardless of which classification we accept, Schwab states that the common feature of all new technologies is the pervasive power of digitization and information technology (15).

### Dark side of the Industry 4.0 technologies

Industry 4.0 technologies are profoundly changing the face of life and work all around the world. They are pushing the factories, organizations, cities, and finally the whole world to the connected smart editions. Despite all the advantages and attractions of new technologies, Schwab (15) directly noticed that we need to recognize and manage the negative impacts of Industry 4.0 technologies, particularly concerning inequality, employment, and labor markets. Ethics and cultural issues are two other vital concerns of Industry 4.0 technologies. Schwab (15) pointed out that Industry 4.0 technologies have adverse and debatable effects. Some of the concerns of scientists about the adverse effects are (17):

1. Increasing psychological distraction,
2. Identity theft,
3. Digital footprints,
4. Privacy monitoring by artificial intelligence,
5. Recording, analyzing, and indexing individual psychological characteristics and behaviors resulting in limited privacy of humans,
6. Abundance in real-time information and immersion in information, constantly available (24/7),
7. More complexity of systems and loss of control, and
8. Cyber-attacks on home and work cyber-devices.

Current subdiscipline of ergonomics including physical, cognitive, and organizational ergonomics do not specifically address the virtual identity of human and problems give raised by new modern cyber-technologies. This paper addresses emerging human performance problems due to the interaction of humans and Industry 4.0 technologies and justifies the need for a new approach and name for the ergonomics of the Industry 4.0 era.

### Cybergonomics: Definition and scope

One year before Murell redefined the word ergonomics, Norbert Wiener, in 1948, introduced a new science for studying control and communication in a living organism and the machine. He coined a new name for his theory called Cybernetics. It was originally taken from the Greek word kybernetes means skilled in steering or governing (18).

Cybernetics is concerned with the comparative study of automatic control systems such as the nervous system and brain and mechanical-electrical communication systems. Cognition, artificial learning, convergence, adaptation, efficiency, efficacy, connectivity and communication, and social control are studied in this science. Since then, the prefix cyber has been used to refer to concepts relating to the internet or the use of modern or advanced systems and technologies. In this context, cyber is used as a synonym for the following words and concepts: computerized, electronic, networked, virtual, mechanized, robotic, high-tech, online, digital, automatic, automated, programmed, on-screen, crypto, technological, multimedia, connected, social, accessible, and real-time. Therefore, the prefix cyber has a wide range of meanings that are mostly related to digital technologies. Accordingly, many familiar words with cyber prefixes are combined today to indicate their digital nature. A leading cyber-related portmanteau is a cyborg, made of words cybernetics and organism. This word was coined in the 1960s and referred to human-machine mixture and interaction (19). Humans with implanted medical devices like artificial cardiac pacemakers are an example of cyborgs. Advanced external devices attached to the human (interactive-exoskeleton) are new examples of the cyborg concept. Cyber-life, cyber-work, cyber-security, cyber-law, and cybercommerce are other mixed words made by cyber prefix. It can be said that many sciences are upgrading to their cyber editions. Most of them have a new name, which distinguishes them from the classic edition.

On this basis, I suggest a new portmanteau called Cybergonomics. A new word composed of prefix “cyber” and word “ergonomics” that their “er” at the end and beginning of each have been merged to one. This word addresses the ergonomic aspects of human cyber-life and cyber-work. This portmanteau based on a search done by Google^®^ on 3 March 2021, when I (as Secretary of the Board of Iranian Ergonomics Society) used this word for the first time in a lecture in the 3^rd^ International Iranian Ergonomics Webinar (March 3-4, 2021, Shiraz, Iran), was not proposed and defined by any other person (20).

Cybergonomics, in its simple meaning, refers to the ergonomics of advanced cyber-technologies. It combines the cyber-science capabilities to address the ergonomics goals. However, in a broader definition, cybergonomics is the study of human performance in interaction with cyber-technologies to optimize individuals' safety, productivity, and health. In other words, the study of the benefits and challenges of emerging technologies in the Industry 4.0 to adapt these technologies to humans' capabilities and physical, mental, and spiritual limitations in the living and working environment will be within the scope of cybergonomics. Cybergonomics wants to integrate cyber-related ergonomic research and knowledge. Table 1 uses Dempesy et al.'s structure to provide a detailed definition of cybergonomics.

**Table 1:** Definition elements of cybergonomics based on six guide word structure.

**Guide word**	**Ergonomics (Dempsey et. al.)**	**Cybergonomics**
Who	Human, People, User, Person	Accounts (human cyber-identity; ID and passwords) Tele-users
What	System, Machine, Equipment, Product, Technology	Cyber-technologies Human biologic big data Cyber-regulations and standards Connected-devices and machines
How	Engineering, Designing, Applying, Studying, Optimizing	Advanced data analytics Cloud computing Artificial Intelligence Machine learning Smart/new materials
When/Where	Environment, Work, Life	Cyber-job and duties Cyber-life and cyber-home Cyber-organization and Cyber-office Cyber-entertainments and leisure
Goal	Safety, Comfort, efficiency	Safety and health (cyber-fatigue, burnout and injuries, cyber-syndrome) Cyber-safety (cyber-attacks and cyber-accidents) Cyber-comfort (cyber-privacy and discomfort, ethics in cyberspace) Cyber-efficiency and productivity

Ergonomics emerged as a multidisciplinary science that means ergonomics and its subdisciplines use other sciences such as physiology, anatomy, engineering, psychology, management, and system design to achieve their goals. Cybergonomics introduces cyber-science as the newest member of the list. Cyber-science specialists cooperate with ergonomists and other experts to overcome the emerging problems of fitting the task to the human.

In the continuation of this paper, the reasons for introducing a new name for the ergonomics of the new industrial age are justified. Nevertheless, before that, a summary of the characteristics and milestones of ergonomics and the industrial revolutions is summarized in Table 2.

**Table 2:** Some milestones of industrial revolutions and ergonomics.

**Ergonomics/human factors milestones**	**Years**	**Industrial Revolutions characteristics and milestones**
Emerging: - Cybergonomics - Neuroergonomics	2000–	4^th^ Industrial Revolution (Industry 4.0) - Digitalization - Cyber physical systems - Wireless Hyper-connected world - Digital transformations - Personal connected devices - Advanced data analytics - Quantum computing - AI technologies - Full automation - Industrial IoT
Developing: - Systems ergonomics (Macroergonomics) - Cognitive ergonomics - Physical ergonomics	1969–2000	3^rd^ Industrial Revolution (Industry 3.0) - Automation - Invention of the Internet - Discovery of nuclear energy - Automation and digitization through the use of electronics and computers - Telecommunications - Partial automation
- Frederick W. Taylor's scientific management (1911) - Frank and Lillian Gilbreth's time and motion study - Murell proposed Ergonomics as an independent scientific discipline (1949) - Publishing the Journal of Ergonomics Starts (1957)	1870–1969	2^nd^ Industrial revolution (Industry 2.0) - Electrification - Discovery of electricity and oil - Invention of the telephone - Expansion of cross-national railroads - Mass-production - Assembly-lines and heavy machinery Pzowered by electricity
- Proposing of word Ergonomji by W. Jastrzebowski (1857)	1760–1870	1^st^ Industrial revolution (Industry 1.0) - Mechanization - Mechanization of the textile and mining industry - Invention of the telegraph - Steam power and engine

## Justification for the new name

Industry 4.0 technologies have led to fundamental changes in human lifestyle and work. For example, in the last 10 years, jobs have been created around the globe that did not exist before. In turn, this issue has changed the relationship between technology and employment. Accordingly, traditional definitions of terms like job, workplace, employer, employee, labor relations, and organization within their old definitions cannot describe the existing conditions. It can be said that the nature of these words is changing. Therefore, there is an increasing need to redefine these words. Also, the introduction of new technologies into the workplace and human life has caused the old ergonomic standards not to meet the new conditions. Therefore, a new set of ergonomic standards related to new technologies is emerging. In the rest of this section, this paper will explain more detail.

### The changing nature of work and related words

#### Jobs and lifestyle

Digital technologies and global communication infrastructure significantly change the traditional concepts of work and payment, enabling the emergence of new types of jobs extremely flexible and inherently transient (the so-called on-demand economy). While these new jobs allow for people to enjoy more flexible working hours and might launch a new wave of innovation in the job marketplace, they also raise significant concerns with regard to the reduced degree of protection in the context of the on-demand economy, where every worker has essentially become a contractor, who no more extended benefits from job security and longevity (15).

Industry 4.0 has been creating new jobs for several years, and people are already being employed in positions that did not exist 10 years ago. According to a recent survey about the impact of Industry 4.0 on jobs creation within the small and medium-sized enterprises in Slovakia, results have shown that new technologies will increasingly displace physical labor in particular, and emerging jobs will put ever-increasing demands on human intellect (21). This shows that the intellectual demands of cyber works push the workers to adapt their capabilities to the requirements of new technologies. New skills are demanded of operators to interact with cyber-physical systems and robots (22). Gallo and Santolamazza (22) argued that the changes accrued due to Industry 4.0 technologies and digitalized environment among maintenance operators. They found that the maintenance operator should be able to find relevant information and predict events by proper use of big data analytics and the ability to interact with computers, digital databases, and robots. Finally, the ability to rapidly adapt his/her skills to innovations is also strongly demanded. Cybergonomics should identify new occupations and conduct ergonomic research on them.

#### Workforce and worktime

The concept of workforce and worktime is profoundly changing in the modern world. Hyper-connectivity is the core driving force. Millions of people worldwide are connected *via* small smart portable devices. They can be accessed 24/7 by employers. The word netizen has been coined as a new portmanteau of the Internet and citizen to refer to people who spend most of their time in online communities and the Internet (23). Ning et al. (24) reported the rapid growth of netizens in the period 2005–2016. Although this advantage increases work speed, the classic concept of workforce and worktime is gradually outdated. Ertel et al. (25) reported a steady growth of non-standard work contracts in advanced societies. New Ways of Working (NWW) is a type of work organization characterized by temporal and spatial flexibility, often combined with extensive use of information and communication technologies (26). Communication technologies have made it increasingly feasible for employees to stay connected to work when not in the office. While some findings indicate that being connected after regular work hours does not necessarily lead to changes in psychosocial work characteristics, well-being, or job-related outcomes (26), but other evidence shows that communication technologies after work hours were associated with the employee's work-to-life conflict and reporting health problems (25, 27, 28). The health effect of accessibility off-the-job and the lost line between working hours and employees' time off should be more investigated.

On the other hand, Internet-based jobs have created a large group of employees, so-called high-tech freelancers, who do not need to be in their offices to do their jobs. The spread of the idea of job creation through entrepreneurship has also greatly influenced the increase in the population of freelancers in advanced and developing economies. They usually do their job duties in their home offices. This, in turn, has made working hours flexible and changed from daily or routine shift work to deadlines. Besides, this may lead to the gradual loss of the boundary between work and life (29). These conditions are suspect in the occurrence of health problems (25). Traditional regimes of shiftwork will not be applicable for this group of workers, and new paradigms of time-work studies and even physiological studies are needed. Application and software developers and media workers such as writers and journalists are growing groups of freelance workers. Determining the legal aspects of the safety, ergonomics, and occupational health of new technologies freelancers is an interesting issue that falls within the scope of cybergonomics.

Another interesting issue about the workforce is the generational cohorts. Generational cohorts refer to people born in the same period. It is assumed that each generation has a similar personality and behavioral characteristics affected by the technological, cultural, and economic situation of the time they were born (30). The classification of generational cohorts follows the same logic as the classification of industrial revolutions. Classification of generations are different, however, Bencsik et al. (31) identified six alive generations of people including Veteran generation (1925–1946), Baby boom generation (1946–1960), X generation (1960–1980), Y generation (1980–1995), Z generation (1995–2010), and Alfa generation (2010+). They described the workplace behavior and personal differences of two digital generations of Y and Z. Y generation were the first waves of the digital generation born into the world of technology. Nevertheless, the Z generation is a generation born at a time when the Internet and advanced communication technologies dominated. Hence, this generation is also referred to as “Internet Generation,” “digital natives,” and “iGeneration” (31). Studying the behavioral differences of generational cohorts in modern organizations and workplaces is a growing field of research in cybergonomics. Aging and gender-related issues for the workforce in the Industry 4.0 era will also be among the research areas in cybergonomics.

#### Workplace and automation

Workplaces, including industrial and non-industrial organizations, have also undergone many changes in advanced industries. 3D printing, robotics, and automation are the core driving force to deliver the industry to the next generation.

3D printers have made it possible to create a new ergonomic design concept of hand tools, personal protective equipment, and workstations. This technology makes it possible to design and produce fit goods for individuals. Semiautonomous and full-autonomous systems have led to a profound change in the relationship between humans and systems. Endsley discussed the Level of Automation (LOA) taxonomies presented by researchers. She presented a human–autonomy system oversight model to mention the key aspects of autonomy design. In this model interchanging role of humans and machines for tasks like the decision, selection, monitoring information, filtering information, information selection, action selection, and action implementation are argued. She concluded that overcoming the automation conundrum in modern system design will be pretty challenging. She noticed that the system designers consider the characteristics of emergent systems, including workload, engagement, and complexity, to keep the human operator informed and able to interact with the system effectively and safely (32).

Human-Robot Collaboration (HRC) is one of the leading topics of research for Industry 4.0 technologies. Collaborative robots (cobots) are advanced technologies used to assist operators in performing physical activities in cyber-physical production systems. This technology combines basic human capabilities with the intelligent capabilities of machines to make things easier. Gualtieri et al. (16) explored emerging research areas related to safety and ergonomics in the interactive robotics industry in a systematic review. They found that cognitive, organizational, and physical ergonomics studies were rising in the Industry 4.0 technologies, particularly interactive robotics. This means that the requirements created by these technologies have required even previously known areas to review information and knowledge. They concluded that research on interactive robotics ergonomics, although emerging, has not yet reached maturity (16).

In a recent study, Longo et al. (33) investigated employees' changing roles and responsibilities in the oil and gas industry and bibliographically examined the amount, type, and manner of papers in which ergonomics and the Industry 4.0 were integrated. They tried to make a clear connection between the Industry 4.0 literature and ergonomics in the oil and gas industry. In addition, they found that researchers have focused on the impact of simulator-based training in many previous studies to improve process safety. In contrast, little research has been published on the use of cognitive solutions and augmented reality and virtual tools, intelligent failure detection systems, and alarm management. Their study recommended further studies in human-automation symbiosis and socio-technical systems due to the excessive novelty of these systems (33). Their comprehensive review of the literature revealed the lack of specific studies on the application of Industry 4.0 technologies such as Augmented Reality-enabled Operations, Virtual Reality-based training, Intelligent Fault Detection, Predictive Maintenance and Alarm Management, Human-Machine Interfaces for Process Control.

#### Organization, employer, and labor relations

The relationship between technological changes and labor relations is a key point in modern Industry 4.0 based organizations. Caliş Duman and Akdemir (34) showed the positive effects of Industry 4.0 technologies on organizational performance based on eight criteria: profitability, sales, production amount, production amount per capita, capacity utilization rate, production speed, product quality, and costs (34). However, Rana and Sharma (35) argued that technology has completely re-invented the way employees engage with their organizations. They reported the results of research, which revealed that 61 % of businesses have already implemented AI initiatives in 2017, with 71 % having an innovation strategy to push new technologies across organizational functions. They proposed a conceptual framework to show the key sources of changes forced by Industry 4.0 technologies and the transforming role of human resource management transforming role in coping with new situations (35). While the concept of labor and production is changing in the new age, old labor relations cannot be responsible for new net-base situations (36). I think the concept of netployees (instead of employees) and netployers (instead of employers) and their relationship will be heard and studied more in future studies.

New types of work contracts between workers and employers have affected the labor relationship between them. These, in turn, push the organizations to reconstruct their old structures and procedures. Human resource management paradigms are changing in new organizations. Spatial dissociation between worker and employer leads to a mental and emotional disconnect between human and his/her job. New strategic approaches for holistic human resource management are needed in modern organizations to cope with knowledge and competence challenges related to new technologies and processes of Industry 4.0. Human resource management also faces the issue of generational cohorts whose personality and work characteristics are different from older generations (37). This is another interesting subject of research in cybergonomics.

Social networks have created new conditions of opportunity and threat for organizations. The patterns of organizational behavior are changing because social networks have provided sharing thoughts between personnel (38). Social networking has made it possible for employees of an organization to have a virtual twin to share their opinions. It has created a new group of people called influencers who play an essential role in creating informal organizations within formal organizations and conducting public opinion. Landis (39) reviewed the relationship between personality and social networks in organizations. As a result, the social network behavior of employees is a new challenge of macroergonomic problems in the cyber era.

In the cyber world, privacy monitoring is a severe human challenge in the workplace and life. Ergonomics has not yet entered into this critical issue and its effect on humans' mental and physical health. Industry 4.0 technologies can record and analyze all the movements and activities and even the thoughts of operators. These analyses can effectively improve system performance but do operators and human users always want to be so monitored? Gualtieri et al. (16) explicitly conclude that humans may not be willing to accept and trust advanced cyber-physical systems without developing ergonomic knowledge in the field of cognitive and organizational for new situations. They suggest that future research should be conducted focusing on operator emotional stability, well-being, and human-centered design, social and psycho-physical interaction with cyber-physical systems (16).

#### Training and empowerment

Hecklau addressed the new requirements of competencies among employees of modern organizations. He concluded that due to the continuous automation of simple manufacturing processes, the number of workplaces with a high level of complexity would increase, which results in the need for a high level of education of the staff with special empowerments in data analysis science. The challenge is to qualify employees to shift their capacities to workplaces with more complex processes and ensure the retention of jobs in changing working environments (37). Schwab reported the results of a survey for the future jobs in the World Economic Forum shows that complex problem solving, social, and systems skills will be far more in demand in 2020 when compared to physical abilities or content skills (15).

### Emerging adverse effects of modern technologies

Cyber-related disorders are modern health problems of the Industry 4.0 era. Cyber-syndrome is the name proposed for these disorders and offers the physical, social, and mental disorders that affect human beings due to excessive interaction with cyberspace. Ning et al. (24) has provided a list of these disorders, some of them including cyberchondria, nomophobia selfities, phantom vibration syndrome, internet addiction syndrome, text neck syndrome, and texting numb. They divided the formation of cyber-syndrome into four stages and classified it into three distinct classes, including (i) physical disorders (poor posture, radiation exposure, tactile sensation, and asymptomatic carrier); (ii) social disorders (social anxiety, social hostility, and social tepidity); and (iii) mental disorders (behavioral disorders, habits disorders, mood disorders, and delusional disorders) (24). Other problems like cyberbullying are also reported (40, 41).

### Emerging new class of ergonomic standards and regulations

Industry 4.0 technological equipment such as interactive implants, internet-connected wearables has revealed the growing need for new solutions, including ergonomic standards, regulations, and evaluation techniques. Following issues are suggested options for new ergonomic standards: teleworking, interchangeable identities resulting from online and off-site human interactions, cyber-job burnout, cybergonomic ethics code, and hyper-connectivity. I believe that ergonomic ethical codes will be of great importance in the ergonomics of Industry 4.0.

It seems that the initial steps to develop ergonomics standards for Industry 4.0 have begun. For example, International Standards Organization (ISO) recently published ISO/TR 9241-810:2020 titled “Ergonomics of human-system interaction—Part 810: Robotic, intelligent and autonomous systems” (42). It should be noted that this document is not yet an ISO standard. Because the letters TR indicate that this document is a Technical Report. These types of ISO deliverables will transform into an ISO standard document after carefully reviewing its functions and further studies on details over time. This indicates that the ergonomic standards of Industry 4.0 technologies are in their early stages.

**Figure 2:**
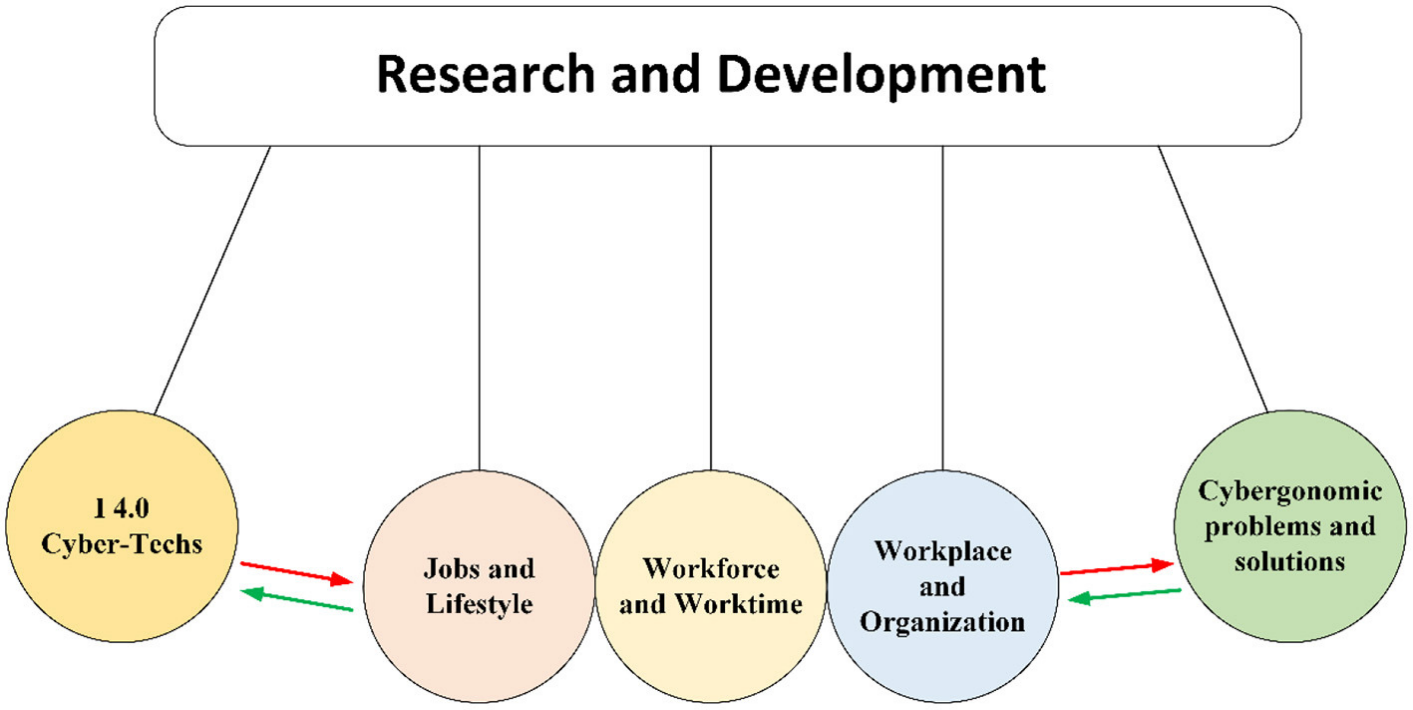
Reciprocating interaction of Industry 4.0 technologies and cybergonomic problems and solutions (a Newton cradle metaphor).

In summary, Figure 2 shows the interaction between Industry 4.0 technologies on concepts like work, workforce, and organization and its role in schematically creating cybergonomic problems and solutions.

## Outline of research areas in cybergonomics

Research on cybergonomics is ongoing, and a large body of related literature is accessible on the Internet. The remarkable point of these studies is that many papers are published in engineering computer science journals such as “Robotics and Computer Integrated Manufacturing,” “Procedia Computer Science,” “Procedia Engineering,” “Technological Forecasting & Social Change.” This suggests that the cyber aspects of these studies are of interest to their authors and computer science journals.

**Figure 3:**
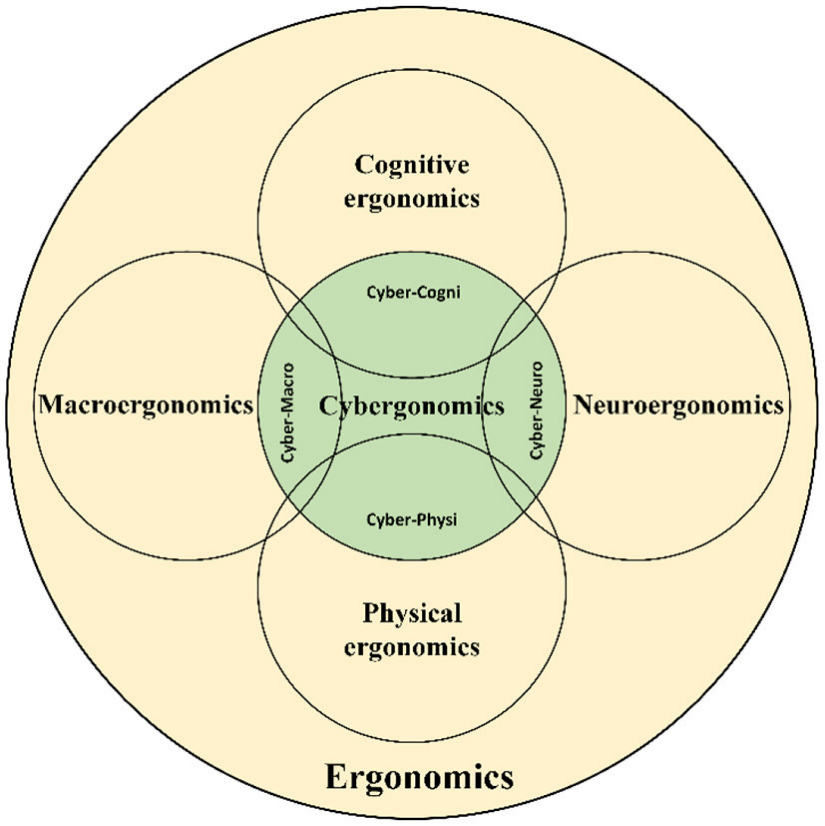
Independent and joint research framework for cybergonomics.

Cybergonomics can have its own research topics and collaborates with other subfields of ergonomics to consider the cyber aspects. Figure 3 schematically shows a general framework for research on cybergonomics. This scheme illustrates the independent and joint areas of research of cybergonomics with existing subdisciplines of ergonomics. Generally, research directions of cybergonomics are related to four theotrical and applied Issues:

- Theoretical studies on redefining the ergonomic values such as safety, comfort, and efficiency. in Industry 4.0 work and organizations.
- Theoretical studies to conceptualization of cybergonomics researches for Industry 5.0.
- The possibilities that Industry 4.0 technologies provide to improve human performance.
- The challenges that Industry 4.0 technologies create for human performance.

In the following, I have speculated on possible research directions.

### Independent fields of research in cybergonomics

How are ergonomic goals and values defined in the Fourth Industrial Revolution? A new look at the commonly used words in ergonomics such as safety, health, comfort, and efficiency, productivity, and teamwork is essential. I mentioned some of these words in Table 1. This is an independent area of research in cybergonomics.

Development in sensors technology and telecommunication is leading to the development of much creative technological equipment. For example, wearable technological gadgets are rapidly emerging and introduced to people's safety and health at work (43, 44). However, the ergonomics aspects of such devices are not examined before commercialization. Hence, extensive cybergonomic research must be done on the design and use of these devices. The failure of Google Glass was one of the most prominent examples of the neglect of cybergonomics. Rajendran et al. (45) recently criticized the idea of equipping workers with wearable technology for real-time monitoring of the workers and eliminating the potential hazards from the workplace. Non-wearable modern devices for industrial and non-industrial use originated from additive manufacturing, smart materials, and I4.0 generation of robotics can also be cybergonomically researched.

Developing modern wearable and non-wearable technologies for ergonomic purposes (cybergonomic devices and assistants) and addressing their ergonomic adverse effects are two main lines of applied research in cybergonomics.

### Joint fields of research in cybergonomics

In this paper, I have already speculated and suggested some new joint research topics to explain the changing nature of jobs, the workforce, and workplaces. However, Table 3 shows some possible research areas for four combinations of cybergonomics with existing subdisciplines of ergonomics.

**Table 3:** Some possible joints and opportunities for research in cybergonomics.

**Joint areas of research**	**Possible topics and opportunities**
Cybergonomics and physical ergonomics	- Big data analysis of anthropometric data - Synthetic biology and physical performance - Synthetic biology and cyber-exoskeletons and musculoskeletal disorders - Adaptive and real-time methods for task scheduling, movement planning and control methods - Cyberspace and emerging musculoskeletal disorders - …
Cybergonomics and cognitive ergonomics	- Cognitive performance of Z generation - Full-automated systems and emotional dissociation - Cognitive aspects of collaborative robotics - Cybergonomic devices and cognition - Cybergonomics and cyber-identity (accounts and passwords) - …
Cybergonomics and macroergonomics	- Privacy and cyber-identity in cyber-organizations - Cyberbullying and organizational protection - Worktime schedules in cyber-organizations - Personnel relations and cyber-organizations - Job burnout and off-the-job in cyber- organizations - Job security and Industry 4.0 technologies - IoT and personal productivity - …
Cybergonomics and nueroergonomics	- Augmented reality and brain performance - Virtual reality and brain - Cloud computing, brain and behavioral data and functions - …

## Discussion

Previous Industrial Revolutions changed the work and lifestyle. This, driven the science to provide new solutions for new problems give raised by living and working with new technologies. In this paper, first, the timeline and emergence of ergonomics and its subsequent various subdisciplines was reviewed from this point of view. Then, the name cybergonomics proposed and defined as a new subdiscipline of ergonomics to cover the emerging ergonomic studies related to Industry 4.0 technologies. At last, the need for this new subdiscipline of ergonomics is argued and justified.

New technologies of Industry 4.0 have created many opportunities and threats for the superior goals of ergonomics, such as reducing errors, increasing safety, increasing comfort, increasing productivity, and improving human-machine interaction (46). However, classic subdisciplines of ergonomics and their common definitions do not specifically address the emerging aspects of life and work in fourth Industrial Revolution (8, 16).

As a simple definition, physical ergonomics deals with human anatomical, anthropometric, physiological and biomechanical characteristics; and cognitive ergonomics is concerned with mental processes of human and their interactions with other elements of a system (7). Finally, Organizational ergonomics is concerned with the optimization of socio-technical systems, including their organizational structures, policies, and processes (7, 47). In fact, they refer to physical, cognitive, and organizational identity of human and its relationship with real world. However, none of these definitions recognize and identify the cyber identity of human and virtual word. Thus, this new identity and new world should be researched in a separate subdiscipline named cybergonomics.

As the speed of data transfer improves from one generation to higher one (4G to 5G and 6G), it is also possible to implement new ideas in Industry 4.0 technologies. New technologies are pushing businesses toward an on-demand economy (35). Evidence of redefinition of key words in ergonomics including work, workforce, worktime, workplace, organization, employer-employee relations was presented and argued in the body of paper. New responsibilities and roles induced by new technologies are expanding (33). Number of netizens, netployees and netployers are increasing exponentially (23, 24, 35, 36). Many newborn jobs or new editions of old jobs require to significant qualifications and skills on working in cyber-space (21, 28, 29, 34). In the body of the paper, I noticed some recent studies that concluded the extensive need for further and future studies on the ergonomic aspects of the interaction of human and cyber-space. For example, the adaptation of employers and employees to cyber-technologies to promote their selves to a cyber-physical system is a serious ergonomic concern (22).

Also, new health problems associated with Industry 4.0 technologies are spreading (25, 26). Various types of cyber-syndrome have increasingly spread in recent decade (24). Hence, many applied areas of Cybergonomic studies can be predictable and now is research under various scientific disciplines. As I referred in this paper, many ergonomic researches on Industry 4.0 technologies currently are reviewed and published in computer science and other applied science journals (22, 33, 34, 43, 44). As an example, application of the theory of cybergonomics to understand the interaction of implanted technologies with human cognition and physical performance fall within the Cybergonomic studies. In this regard, the study of ergonomic aspects of human tele-communication through brain signals is one of the exciting fields of cybergonomics studies. Also, identifying and characterizing new adverse health problems of using Industry 4.0 technologies such as smart textiles and wearable internet are another example of applied research in cybergonomics.

Nowadays the concept of Industry 5.0 has been introduced and is developing to make a peace between Industry 4.0 technologies and human being as the live element of system. Industry 5.0 places the wellbeing of the worker at the center of the production process and uses new technologies to provide wellbeing beyond jobs and growth to become a resilient provider of prosperity (46). This concept, with the aim of correcting the characteristics and situations brought by the technologies of Industry 4.0, tries to bring the workforce back to the work environment and provide the possibility of customizing the work and life environment more than before (48). From this point of view, this concept is sometimes called society 5.0, because it tries to increase the communication and interaction of humans with each other in the society by removing the problems caused by the technologies of Industry 4.0. Thus, industry 5.0 is aimed at supporting—not superseding—humans. Cybergonomics as a new subdiscipline of ergonomics is in line with the goals of Industry 5.0. Cybergonomics can help Industry 5.0 to achieve its goals to protect human from adverse effects of new technologies and provide necessary rules and adaptations to better interaction of workforce and high-technologies.

In conclusion, it can say that cybergonomics try to gather various researchers' efforts under one umbrella to proliferate our insight on how we make a peace between goals of ergonomics and Industry 4.0 technologies.

There are several limitations to my research. First, this paper is based on theoretical review of literature and it reflects a perspective article based on personal expectations and viewpoints. Second, it was based on available information regarding Industry 4.0 technologies such as research articles, reports and books. Many pioneer technologies of Industry 4.0 are not reflected in research articles and academic publications. Finally, it should be noted that no literature about cybergonomics is now present in databases as this word is a new portmanteau to describe a new subdiscipline of ergonomics. Thus, running a systematic review for this concept was impossible. I tried to review the related literature based on keywords which were related to my hypothesis and speculation. Future researches may present empirical analysis of all the ideas discussed in the paper.

## Data availability statement

The original contributions presented in the study are included in the article/supplementary material, further inquiries can be directed to the corresponding author/s.

## Author contributions

All the relevant activities including conceptualization, search and review, and writing the manuscript is done by MP.

## Conflict of interest

The author declares that the research was conducted in the absence of any commercial or financial relationships that could be construed as a potential conflict of interest.

## Publisher's note

All claims expressed in this article are solely those of the authors and do not necessarily represent those of their affiliated organizations, or those of the publisher, the editors and the reviewers. Any product that may be evaluated in this article, or claim that may be made by its manufacturer, is not guaranteed or endorsed by the publisher.
